# Impact of auditory feedback alterations in individuals with stuttering^☆^

**DOI:** 10.1016/j.bjorl.2019.08.005

**Published:** 2019-10-03

**Authors:** Michele Fiorin, Eduarda Marconato, Talissa Almeida Palharini, Luana Altran Picoloto, Ana Cláudia Figueiredo Frizzo, Ana Claudia Vieira Cardoso, Cristiane Moço Canhetti de Oliveira

**Affiliations:** Universidade Estadual Paulista Júlio de Mesquita Filho (Unesp), Faculdade de Filosofia e Ciências, Programa de Pós-Graduação em Fonoaudiologia, Marília, SP, Brazil

**Keywords:** Speech-language pathology, Speech disorders, Stuttering, Hearing, Feedback

## Abstract

**Introduction:**

Electrophysiological evidence has reinforced the hypothesis that stuttering is associated with a deficit in modulation of the cortical auditory system during speech planning, contributing to an inefficient auditory feedback monitoring and, consequently, resulting in disfluencies.

**Objective:**

To verify the impact of auditory feedback modifications on the spontaneous speech of individuals with stuttering.

**Methods:**

Sixteen individuals, of both genders, aged 8–17 years and 11 months, with a diagnosis of persistent neurodevelopmental stuttering, were divided into two groups: Moderate Stuttering Group and Severe Stuttering Group. The testing procedures consisted of three stages: collection of identification data, audiological assessment and fluency evaluation of spontaneous speech in four auditory feedback conditions (non-altered, delayed, masked and amplified). The speech sample obtained in the non-altered feedback was considered the control; the others were considered as modified listening conditions.

**Results:**

Regarding the stuttering-like disfluencies, a statistically significant difference was observed in the intragroup analysis of the Moderate Stuttering Group between non-altered and masked auditory feedback (*p* = 0.042), as well as between non-altered and amplified (*p* = 0.042). There was a statistically significant difference in the Severe Stuttering Group for all auditory feedback modifications in relation to the non-altered (delayed *p* = 0.012, masked *p* = 0.025 and amplified *p* = 0.042). There was also a reduction in flows of syllables and words-per-minute in the Moderate Stuttering Group for the delayed auditory feedback, as compared to non-altered (*p* = 0.017 and *p* = 0.025, respectively).

**Conclusion:**

The effect of delayed auditory feedback was favorable for the Severe Stuttering Group, promoting speech fluency. The conditions of masked and amplified auditory feedback resulted in speech benefits in both groups, decreasing the number of stuttering-like disfluencies. The speech rate was not impaired by any listening condition analyzed.

## Introduction

Stuttering is a complex multidimensional neurodevelopmental disorder with a widely investigated neurobiological basis. There is scientific evidence that individuals who stutter have structural and functional abnormalities in the areas responsible for oral language. The most common findings involve the reduction of white matter along the portions of the arcuate /superior longitudinal fascicle in stuttering children and adults.^1^ Neuroimaging studies have also demonstrated reduced connectivity in areas of the brain responsible for processing time and rhythm^2^; atypical development of the auditory-motor neural network and the thalamus-cortical basal ganglia^3^; primary deficit in the left hemisphere speech network, specifically involving the lateral premotor cortex and the primary motor cortex.^4^

Recent studies have shown relevant results about the auditory regions and their connections with brain areas in speech production.^3^, ^5^, ^6^ Electrophysiological evidence has reinforced the hypothesis that stuttering is associated with a deficit in the cortical auditory system modulation during speech planning, contributing to the inefficient monitoring of auditory feedback and, thus, resulting in disfluencies.^7^

Aiming to clarify the important role of hearing in the continuity of speech flow, many studies have contributed with clinical implications in stuttering diagnosis and therapy. Among these implications are auditory feedback alterations, which are being used as therapeutic resources to promote fluency in stuttering individuals.

The basal ganglia and cerebellum play a significant role in speech production under the auditory feedback alterations. According to the dual pre-motor model,^8^ there are two systems involved in the planning and performance of speech movements: the lateral system, comprising the lateral premotor cortex and the cerebellum, responsible for the motor response to external sensory stimuli; and the medial system, consisting of the basal nuclei and the supplementary motor area, dominant in spontaneous speech, without alterations in auditory feedback. The model suggests that the supplementary motor area is responsible for the motor programming of each speech segment and that the basal ganglia help in this process, providing internal temporal clues to facilitate movement onset.

According to this perspective, stuttering would be caused by a disturbance in the medial system, especially in the region of the basal ganglia.^9^ This theory justifies an increase in fluency under auditory feedback alterations, since individuals who stutter compensate for this deficit with an external timing mechanism, which is supported by the cerebellum and premotor cortex.^9^

Auditory feedback refers to the speech sounds perceived by the speaker's own auditory system during oral emission and it is a component that helps with the speech movement control mechanisms.^10^ When a sudden irregularity occurs in a specific acoustic parameter of auditory feedback, fluent speakers can instantly correct the error in their oral production, while individuals who stutter have shown a weaker-than-normal compensation when experiencing these occurrences.^10^ These findings indicate that individuals who stutter cannot auditorily compare the desired speech movements with the actual movements as effectively as fluent speakers.^11^

Delayed auditory feedback was the most commonly indicated condition for individuals who stutter.^12^, ^13^, ^14^, ^15^, ^16^, ^17^ However, there are other types of auditory feedback alterations, such as masked and amplified auditory feedback, which have been scarcely investigated in this population.

Studies carried out with delayed auditory feedback (DAF) in individuals who stutter are different regarding the analyzed variables. Most of them observed the effect of DAF on the frequency of disfluencies,^12^, ^13^, ^14^, ^15^, ^16^, ^17^ while others assessed changes in speed^12^, ^18^ or naturalness of speech,^14^, ^19^ and few have investigated the impact on stuttering severity.^13^, ^15^

In this context, there is no consensus on the results of delayed auditory feedback studies in stuttering individuals. Some studies have shown benefits in fluency,^20^, ^21^, ^22^ while others concluded there is a diversity in the results found in this population.^17^, ^23^, ^24^, ^25^

The studies with masked auditory feedback in stutterers have shown a reduction in the main manifestations of the disorder, which are the stuttering-like disfluencies.^26^, ^27^, ^28^

Several factors can interfere with the results, such as the type of feedback, age, disfluency typology, and stuttering severity, among others. Researchers tend to believe that the subgroup of individuals with more severe stuttering obtain greater benefits than the ones with a lower degree of severity, regarding both the delayed^17^, ^23^, ^24^ and the masked auditory feedback.^29^

The amplification effects have been studied in other populations, and the researchers have found an immediate reduction in vocal intensity, easier and more stable emission, less tense vocal quality and longer maximum phonation time.^30^, ^31^ Therefore, it is presumed that amplification can also help in the fluency of stuttering speakers.

The assessed scientific literature showed no scientific evidence of which subgroups of stuttering individuals obtained the greatest benefit from these resources. As a consequence, this study aims to increase the understanding of the indication criteria for the use of different auditory feedback conditions in stuttering.

Thus, the aim of this study was to verify the impact of auditory feedback alterations on the spontaneous speech of individuals who stutter.

## Methods

The study was carried out according to the National Health Council (Resolution N. 466/12) and was initiated only after approval by the Research Ethics Committee of Faculdade de Filosofia e Ciências – UNESP/Marília (Opinion N. 0714/2013). All individuals had their participation authorized by signing the Free and Informed Consent Form and the Term of Assent.

A prospective observational cross-sectional clinical study was carried out with 16 native speakers of Brazilian Portuguese, of both genders, aged 8–17 years and 11 months, with a speech-language pathology diagnosis of persistent neurodevelopmental stuttering. The sample consisted of individuals recruited at a school clinic through a service specialized in the evaluation, diagnosis and treatment of fluency and its disorders, who were divided into two groups: the Moderate Stuttering Group (MSG), consisting of eight individuals with moderate stuttering, two females and six males; and the Severe Stuttering Group (SSG), consisting of eight individuals with severe stuttering, three females and five males. The mean age for both groups was 11 years, and the disorder severity was classified according to the *Stuttering Severity Instrument* – SSI-3.^32^

The following inclusion criteria were used for participation in this study: the onset of stuttering must have occurred during childhood (neurodevelopmental); duration of at least 36 months of stuttering-like disfluencies without remission (persistent); minimum 3% of stuttering-like disfluencies^33^; audiometric thresholds within the normal limits (with thresholds up to 25 dBNA at the sound frequencies of 250 Hz–8 kHz); Tympanometry—type A curve (normal mobility of the tympanic-ossicular system); present contralateral stapedial acoustic reflexes (at the sound frequencies of 500 Hz–4 kHz); no history of conduction and/or neurological alterations^34^; and not currently attending speech-language therapy sessions.

The Stuttering Severity Instrument – SSI-3 – was used to classify the severity of stuttering as moderate or severe,^32^ in the non-altered speech sample. The SSI-3 evaluated the frequency and duration of stuttering-like disfluencies, as well as the presence of concomitant physical associated with the disfluencies.

The research procedures consisted of three stages: (1) Collection of identification data; (2) basic audiological assessment; and (3) classification of stuttering severity under the condition of non-altered auditory feedback and evaluation of spontaneous speech fluency under four conditions of auditory feedback (non-altered, delayed, masked and amplified).

At the first stage, the specific clinical history was obtained, in which the parents/guardians of the stuttering individuals were asked orally about the identification data; health history, speech/language and familial problems; complaints and previous history of complaints; and inherent questions about the onset of the disorder, as well as questions related to hearing.

The second stage consisted of the basic audiological evaluation, consisting of pure tone threshold audiometry (250 Hz–8 kHz), logoaudiometry (Speech Recognition Threshold - SRT) and immittance audiometry (tympanometry and acoustic reflexes) to rule out any hearing impairment. The audiometry was performed in an acoustic booth, using TDH-50 earphones and a Grason-Stadler GSI 61 Audiometer, calibrated according to ANSI-69 standards. Thresholds were considered within the normal range when they showed an intensity of 25 dB or lower at all tested sound frequencies (250 Hz–8 kHz).

The SRT was assessed using a list of disyllabic words and should be compatible with the audiometry results (0–10 dB above the tritonal average). For the immittance audiometry, a Grason-Stadler GSI-33 audiometer was used, with a 226 Hz probe. The tympanometry was considered within the normal range when a tympanometric curve A was obtained, indicating normal mobility of the tympanic-ossicular system. Subsequently, the acoustic reflex, ipsilateral and contralateral mode, was assessed, for which the presence of contralateral reflexes was considered as normality (at 500 Hz–4 kHz sound frequencies).^35^

Fluency evaluation was performed at the third stage, in which spontaneous speech samples were collected from each of the individuals under four feedback conditions: non-altered, delayed, masked and amplified. Speech sequence recording was performed in the same way for all participants, providing a two-minute silence interval between each collected speech sample. Moreover, the recordings were carried out with the individual sitting in a quiet environment, with the microphone and headphones adjusted and connected to a computer, using the Fono Tools software (version 1.5 h, CTS Informática). Initially, the speech sample was recorded as usual and, subsequently, it was processed using the software, which modified the auditory feedback under three listening conditions: delayed, masked and amplified.

It is also noteworthy that the speech sample obtained with the non-altered feedback was considered a control condition; the others were considered as modified listening conditions (delayed, masked and amplified feedback).

In the delayed auditory feedback, the recorded speech stimulus was returned to the individual's ear with a 100-millisecond delay through supra-aural headphones. Using this resource, the speakers heard their own voice as a choir effect.^23^ Regarding the masked auditory feedback, a white noise was applied. The intensity used in the masked and amplified auditory feedback ranged from 65 dB to 90 dB, being adopted the intensity referred by the individual as the one with maximum comfort.

After the speech samples were collected under the four auditory feedback conditions, they were fully transcribed, considering a total of 200 fluent syllables for each sample,^36^ and the disfluency events were recorded and coded in the text. The analysis and characterization of the disfluency typology was also performed, according to the following description: Stuttering-like Disfluencies (SLDs): block, prolongation, pause, intrusion, sound repetition, syllable repetition and word repetition — above 3; Other Disfluencies (OD): Interjection, hesitation, revision, incomplete word, phrase repetition and word repetition — up to two.^33^, ^37^

Speech rate was calculated using the flow of syllables per minute (SPM) and flow of words per minute (WPM). The duration of each speech sample was timed, and for this calculation the silent time (pauses and unfilled hesitations) was not discounted, nor the time spent in the production of disfluencies, a methodology proposed by the Fluency Assessment Protocol.^38^ The evaluator's speech was removed from the sample and, subsequently, the total utterance time (TTEe) was measured,^39^ related to the production of the 200 fluent syllables.

The statistical analysis aimed to verify if there were intragroup differences using the Wilcoxon signed-ranks test. To check for intergroup differences, the Mann–Whitney test was applied. For all conclusions obtained through inferential analyses, a significance level of 5% or less (*p* < 0.05) was adopted. Results with statistical differences were highlighted with the asterisk symbol (*).

## Results

The study included 16 individuals of both genders, aged 8–17 years and 11 months, divided into two groups: MSG (Moderate Stuttering Group), consisting of eight individuals with moderate stuttering, two females and six males; and SSG (Severe Stuttering Group), consisting of eight individuals with severe stuttering, three females and five males. The mean age for both groups was 11 years.

**Figure 1:**
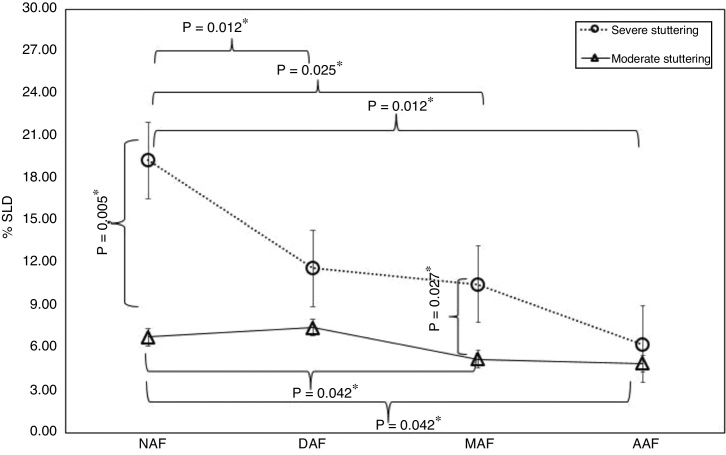
Intragroup and intergroup analysis of the percentage of stuttering-like disfluencies under the different auditory feedback conditions. %SLD, percentage of stuttering-like disfluencies; NAF, Non-Altered Auditory Feedback; DAF, Delayed Auditory Feedback; MAF, Masked Auditory Feedback; AAF, Amplified Auditory Feedback. *Statistically significant values.

In the analysis of the stuttering-like disfluencies, regarding the intragroup results of the MSG, a statistically significant reduction was observed in the speech samples obtained under the conditions of Masked (MAF) and Amplified Auditory Feedback (AAF) compared to the non-altered auditory feedback (NAF). In the intragroup results of SSG, we verified a statistically significant reduction in all auditory feedback conditions (DAF, MAF and AAF) when compared to the non-altered auditory feedback (NAF). The intergroup comparison revealed that the SSG showed a higher number of stuttering-like disfluencies than MSG under the NAF and MAF conditions, with a statistically significant difference (Fig. 1).

In the analysis of the other disfluencies, intra and intergroups, no significant differences were identified in any of the auditory feedback conditions. Regarding the total disfluencies, there was a significant reduction for SSG in the MAF and AAF conditions in relation to the NAF. In the intragroup analysis of the MSG and in the intergroup comparison, there was no significant difference in any of the auditory feedback conditions (Table 1).

**Table 1:** Intragroup and intergroup analysis regarding the percentage of other disfluencies and total disfluencies in spontaneous speech under different listening conditions.

	Other disfluencies								
Listening conditions	MSG				SSG				*p*-value
	$\bar{\chi }$	Min.	Max.	SD	$\bar{\chi }$	Min.	Max.	SD	
NAF	13.81	6.00	25.50	6.21	12.63	5.50	18.50	4,22	0,752
DAF	12.44	9.00	21.50	3.99	14.69	4.50	31.50	8,31	0,316
*p*-value		0.734				0.352			
NAF	13.81	6.00	25.50	6.21	12.63	5.50	18.50	4,22	0,752
MAF	12.50	2.50	26.50	7.84	12.50	6.00	17.50	4,12	0,636
*p*-value		0.624				0.888			
NAF	13.81	6.00	25.50	6.21	12.63	5.50	18.50	4,22	0,752
AAF	10.88	5.50	18.00	4.09	10.75	3.00	15.00	3,73	0,999
*p*-value		0.233				0.176			
	Total of disfluencies								
NAF	21.38	9.00	35.00	8.48	31.94	20.50	49.50	11,29	0,666
DAF	19.94	11.00	29.00	6.28	26.38	8.50	42.50	12,39	0,345
*p*-value		0.528				0.161			
NAF	21.38	9.00	35.00	8.48	31.94	20.50	49.50	11,29	0,066
MAF	17.50	6.00	37.00	11.15	23.06	11.50	34.50	8,35	0,189
*p*-value		0.327				0.036^a^			
NAF	21.38	9.00	35.00	8.48	31.94	20.50	49.50	11,29	0,066
AAF	15.81	8.00	26.50	6.34	17.06	6.50	26.50	6,14	0,752
*p*-value		0.058				0.012^a^			

MSG, Moderate Stuttering Group; SSG, Severe Stuttering Group; NAF, Non-Altered Auditory Feedback; DAF, Delayed Auditory Feedback; MAF, Masked Auditory Feedback; AAF, Amplified Auditory Feedback; $\bar{\chi }$, mean; Min., Minimum; Max., Maximum; SD, standard deviation.

a Statistically significant values.

As for the speech rate, there was a statistically significant reduction in syllable and word flows per minute for the MSG group in the speech sample obtained in DAF in relation to the NAF; and for SSG, there was no significant difference in any of the auditory feedback conditions (Table 2).

**Table 2:** Intragroup and intergroup analysis regarding flow of syllables per minute and words per minute in spontaneous speech under different listening conditions.

	Syllables per minute								
Listening conditions	MSG				SSG				
	$\bar{\chi }$	Min.	Max.	SD	$\bar{\chi }$	Min.	Max.	SD	*p*-value
NAF	131.88	50.00	292.00	77.39	87.25	48.00	169.00	44,56	0,189
DAF	103.25	33.00	245.00	64.59	103.25	49.00	226.00	57,82	0,916
*p*-value		0.017^a^				0.123			
NAF	131.88	50.00	292.00	77.39	87.25	48.00	169.00	44,56	0,189
MAF	138.63	36.00	230.00	66.54	103.63	74.00	136.00	26,15	0,207
*p*-value		0.999				0.401			
NAF	131.88	50.00	292.00	77.39	87.25	48.00	169.00	44,56	0,189
AAF	130.50	50.00	250.00	70.85	111.25	59.00	150.00	38,09	0,599
*p*-value		0.866				0.326			
	Words per minute								
NAF	75.75	31.00	157.00	40.20	51.00	28.00	96.00	25,47	0,155
DAF	62.63	20.00	133.00	35.12	59.13	29.00	133.00	35,26	0,833
*p*-value		0.025^a^				0.293			
NAF	75.75	31.00	157.00	40.20	51.00	28.00	96.00	25,47	0,155
MAF	76.13	22.00	122.00	34.59	60.38	42.00	85.00	17,19	0,343
*p*-value		0.779				0.122			
NAF	75.75	31.00	157.00	40.20	51.00	28.00	96.00	25,47	0,155
AAF	72.63	30.00	130.00	37.23	60.25	28.00	90.00	21,13	0,431
*p*-value		0.674				0.362			

MSG, Moderate Stuttering Group; SSG, Severe Stuttering Group; NAF, Non-Altered Auditory Feedback; DAF, Delayed Auditory Feedback; MAF, Masked Auditory Feedback; AAF, Amplified Auditory Feedback; $\bar{\chi }$, mean; Min., Minimum; Max., Maximum; SD, standard deviation.

a Statistically significant values.

## Discussion

The knowledge of the correct indications for the use of auditory feedback alterations in stuttering is essential to recommend this therapy when it is really beneficial to the individual. The literature has shown contradictory effects regarding these alterations, as well as a scarcity of studies assessing the masking and amplification in individuals who stutter; thus, further studies with more careful methodological designs are required.

An important factor that influences the results of auditory feedback alterations in individuals who stutter is the condition used, with the most frequent one being the delay. Another relevant factor is the severity of the disorder. Although few researchers use the stuttering severity classification and analyze the impact of auditory feedback alterations in homogeneous groups regarding the severity, the results showed that the benefits were greater in individuals with more severe stuttering, both in DAF^17^, ^23^, ^24^ and in MAF.^29^

This study aimed to verify the impact of delay, masking and amplification of auditory feedback on spontaneous speech of individuals who stutter, in two distinct groups regarding the severity of the disorder: one group with moderate stuttering (MSG) and one with severe stuttering (SSG).

Regardless of the fact that some researchers report reduced stuttering with delayed auditory feedback (DAF),^20^, ^21^, ^22^ the results of the present study showed that the impact was positive only for individuals with severe stuttering. Thus, the indication of DAF should be based on a more careful phenotype refinement to avoid the indiscriminate use of this resource in all individuals who stutter.

In addition to the severity of the disorder, the typology of disfluencies may be another criterion to be used when indicating DAF. A recent study of individuals with different stuttering severities showed that the delayed auditory feedback resulted in a reduction of word repetition frequency and articulatory speed, without impairing the flow of information.^40^

The DAF resulted in a reduction in the stuttering-like disfluencies in the SSG, the main manifestation of the disorder,^20^, ^21^, ^22^ and, consequently, a reduction of total disfluencies, promoting speech fluency. This finding corroborated previous investigations that described the effect of DAF as beneficial for individuals with severe stuttering, when compared to those with less severe stuttering.^17^, ^23^, ^24^ Individuals with moderate stuttering responded to DAF differently from individuals with severe stuttering, as the speech rate decreased (syllable and word flow per minute) and there was no change in the number of disfluencies (stuttering-like disfluencies, other disfluencies and total disfluencies).

The increase in the speech fluency promotion of individuals with severe stuttering was not caused by the decrease in the speech rate, disagreeing with a previous study.^12^ The results found in this study corroborate others showing that the decrease in the number of disfluencies is not associated with reduced speech rate.^13^, ^16^, ^17^, ^18^, ^25^, ^26^, ^29^^,^^41^ It is also noteworthy that the reduction in speech rate is not desirable for individuals who stutter, since this characteristic manifests due to the excess number of disfluencies^42^, ^43^, ^44^, ^45^ and/or articulatory slowing down.^46^

The results related to masking (MAF) and amplification (AAF) were positive in individuals who stutter, regardless of the severity of the disorder. The remarkable reduction of SLDs (45.31%) in the SSG, obtained with masking, contributed to a significant decrease of Total Disfluencies (TD) under this listening condition. However, the MSG decreased 22.76% of SLDs and was probably not sufficient to significantly reduce total disfluencies. This finding was consistent with a study by Altrows and Bryden,^24^ In which the authors reported that, when using masking, the most severe stutterers tended to show more efficacy in increasing fluency than the stutterers with other types of severity.

The findings of this study support the fact that the increased fluency of individuals who stutter under the masking auditory feedback is not only associated with a reduced speech rate, but also as a consequence of the modified auditory input.^26^, ^27^, ^29^ Therefore, it is assumed that auditory input processing may be different in individuals who stutter, when compared to fluent individuals.^47^

The assessed literature showed no studies that used amplified auditory feedback in individuals who stutter. Thus, it was not possible to make comparisons between the obtained data and those of other studies, considering the originality of this research.

The intergroup analysis (Moderate Stuttering and Severe Stuttering) showed that the groups did not differ in spontaneous speech during amplified auditory feedback (AAF), either in disfluency frequency or speech rate. The intragroup analysis showed there was a decrease in SLDs in both groups, but the numerical values showed this reduction was greater in the SSG (67.32%) than in the MSG (27.46%).

The comparison between the group with moderate stuttering (MSG) and the group with severe stuttering (SSG) under the effect of MAF showed that the only significant difference was observed for SLDs. This fact can be justified, considering that these disfluencies are the main manifestations of the disorder, and the severe group continued to present a larger number of them than the moderate group, since the reduction of these disfluencies occurred in both groups.

Amplification is known to produce an immediate reduction in vocal intensity, an easier and more stable emission, less tense vocal quality and longer maximum phonation time.^30^, ^31^ These factors have possibly contributed to promote the fluency of stuttering individuals, with a presumed reduction in muscle tension and an increase in the maximum phonation time.

Even though the findings are relevant, one of the limitations of this study is related to the level of intensity used for the masked and amplified auditory feedback, considering this level ranged from 65 dB to 90 dB, and was determined according to the maximum comfort level reported by the individual. Another limitation is related to the fact that the central auditory processing evaluation was not performed, considering the high prevalence of this auditory processing disorder in individuals who stutter^34^, ^48^, ^49^, ^50^; for this reason, the inclusion of this assessment is recommended in future studies.

Despite these limitations, this study provided new information on therapeutic resources available for individuals with moderate and severe stuttering, indicating that the identification of the stuttering subgroup regarding the disorder severity was relevant, as therapeutic strategies vary according to the disorder severity.

The results propose that, for individuals with severe stuttering, the speech therapist use the delayed, masked and amplified auditory feedback to assist in therapy; accordingly, individuals who stutter should also be encouraged, if they feel comfortable, to use these resources in communication situations where they need more speech fluency. For cases of moderate stuttering, however, masking and amplification are recommended. The use of these resources does not replace traditional speech-language therapy to promote fluency, but they are tools that will help with the therapeutic results.

The is a groundbreaking study, as it demonstrates that the resources applied in speech-language therapy should be differentiated for individuals with moderate and severe stuttering.

## Conclusion

The effect of delayed auditory feedback was favorable for the severe stuttering group (SSG), promoting speech fluency. The Masked (MAF) and Amplified (AAF) Auditory Feedback conditions showed spontaneous speech benefits for both groups (MSG and SSG), reducing the number of stuttering-like disfluencies. Speech rate was not impaired by any of the listening conditions analyzed.

## Conflicts of interest

The authors declare no conflicts of interest.

## References

[1] Chang S.E., Garnett E.O., Etchell A., Chow H.M. (2018). Functional and neuroanatomical bases of developmental stuttering: current insights. Neuroscientist.

[2] Chang S.E., Horwitz B., Ostuni J., Reynolds R., Ludlow C.L. (2011). Evidence of left inferior frontal-premotor structural and functional connectivity deficits in adults who stutter. Cereb Cortex.

[3] Chang S.E. (2015). Subtle differences in brain network connectivity in children who stutter. Procedia Soc Behav Sci.

[4] Garnett E.O., Chow H.M., Nieto-Castñón A., Tourville J.A., Guenther F.H., Chang S.E. (2018). Anomalous morphology in left hemisphere motor and premotor cortex of children who stutter. Brain.

[5] Lu C., Long Y., Zheng L., Shi G., Liu L., Ding G. (2016). Relationship between speech production and perception in people who stutter. Front Hum Neurosci.

[6] Daliri A., Wieland E.A., Cai S., Guenther F.H., Chang S.E. (2018). Auditory-motor adaptation is reduced in adults who stutter but not in children who stutter. Dev Sci.

[7] Daliri A., Max L. (2015). Modulation of auditory processing during speech movement planning is limited in adults who stutter. Brain Lang.

[8] Goldberg G. (1985). Supplementary motor area structure and function: review and hypotheses. Behav Brain Sci.

[9] Alm P.A. (2004). Stuttering and the basal ganglia circuits: a critical review of possible relations. J Commun Disord.

[10] Cai S., Beal D.S., Ghosh S.S., Tiede M.K., Guenther F.H., Perkell J.S. (2012). Weak responses to auditory feedback perturbation during articulation in persons who stutter: evidence for abnormal auditory-motor transformation. PLoS One.

[11] Hudock D., Dayalu V.N., Saltuklaroglu T., Stuart A., Zhang J., Kalinowski J. (2011). Stuttering inhibition via visual feedback at normal and fast speech rates. Int J Lang Commun Disord.

[12] Curlee R.F., Perkins W.H. (1973). Effectiveness of a DAF conditioning program for adolescent and adult stutterers. Behav Res Ther.

[13] Sparks G., Grant D.E., Millay K., Walker-Batson D., Hynan L.S. (2002). The effect of fast speech rate on stuttering frequency during delayed auditory feedback. J Fluency Disord.

[14] Stuart A., Kalinowski J., Saltuklaroglu T., Guntupalli V.K. (2006). Investigations of the impact of altered auditory feedback in-the-ear devices on the speech of people who stutter: one-year follow-up. Disabil Rehabil.

[15] Antipova E.A., Purdy S.C., Blakeley M., Williams S. (2008). Effects of altered auditory feedback (AAF) on stuttering frequency during monologue speech production. J Fluency Disord.

[16] O’Donnell J.J., Armson J., Kiefte M. (2008). The effectiveness of SpeechEasy during situations of daily living. J Fluency Disord.

[17] Unger J.P., Gluck C.W., Cholewa J. (2012). Immediate effects of AAF devices on the characteristics of stuttering: a clinical analysis. J Fluency Disord.

[18] Kalinowski J., Stuart A. (1996). Stuttering amelioration at various auditory feedback delays and speech rates. Eur J Disord Commun.

[19] Armison J., Kiefte M. (2008). The effect of Speech Easy on Stuttering frequency, speech rate, and speech naturalness. J Fluency Disord.

[20] Carrasco E.R., Schiefer A.M., Azevedo M.F. (2015). Effect of the delayed auditory feedback in stuttering. Audiol Commun Res.

[21] Ritto A.P., Juste F.S., Andrade C.R.F. (2015). Impacto do uso do SpeechEasy® nos parâmetros acústicos e motores da fala de indivíduos com gagueira. Audiol Commun Res.

[22] Ritto A.P., Juste F.S., Stuart A., Kalinowiski J., Andrade C.R. (2016). Randomized clinical trial: the use of SpeechEasy® in stuttering treatment. Int J Lang Commun Disord.

[23] Andrade C.R.F., Juste F.S. (2011). Systematic review of delayed auditory feedback effectiveness for stuttering reduction. J Soc Bras Fonoaudiol.

[24] Foundas A.L., Mock J.R., Corey D.M., Golob E.J., Conture E.G. (2013). The SpeechEasy device in stuttering and nonstuttering adults: fluency effects while speaking and reading. Brain Lang.

[25] Picoloto L.A., Cardoso A.C.V., Cerqueira A.V., Oliveira C.M.C. (2017). Effect of delayed auditory feedback on stuttering with and without central auditory processing disorders. CoDAS.

[26] Lincoln M., Packman A., Onslow M. (2006). Altered auditory feedback and the treatment of stuttering: a review. J Fluency Disord.

[27] Hampton A., Weber-Fox C. (2008). Non-linguistic auditory processing in stuttering: evidence from behavior and event-related brain potentials. J Fluency Disord.

[28] Ingham R.J., Bothe A.K., Wang Y., Purkhiser K., New A. (2012). Phonation interval modification and speech performance quality during fluency ‒ inducing conditions by adults who stutter. J Commun Disord.

[29] Altrows I.F., Bryden M.P. (1977). Temporal factors in the effects of masking noise on fluency of stutterers. J Commun Disord.

[30] Behlau M., Behlau M (2005). Voz: o livro do especialista.

[31] Coutinho S.B., Diaféria G., Oliveira G., Behlau M. (2009). Voice and speech of individuals with Parkinson´s disease during amplification, delay and masking situations. Pró-Fono R Atual Cient.

[32] Riley G.D. (1994).

[33] Yairi E., Ambrose N. (1992). Onset of stuttering in preschool children: select factors. J Speech Hear Res.

[34] Prestes R., Andrade A.N., Santos R.B., Marangoni A.T., Schiefer A.M., Gil D. (2017). Temporal processing and long-latency auditory evoked potential in stutterers. Braz J Otorhinolaryngol.

[35] Perrucini D.S., Cardoso A.C.V., Moura R.B.O., Lorena M.C.M., Buzzeti P.B.M.M., Oliveira C.M.C. (2017). Effect of delayed auditory feedback on clutter’s speech and reading. Audiol Commun Res.

[36] Ambrose N.G., Yairi E. (1999). Normative disfluency data for early childhood stuttering. J Speech Lang Hear Res.

[37] Pinto J.C.B.R., Schiefer A.M., Avila C.R.B. (2013). Disfluências e velocidade de fala em produção espontânea e em leitura oral em indivíduos gagos e não gagos. Audiol Commun Res.

[38] Andrade C.R.F., ANDRADE C.R.F. (2011). ABFW: teste de linguagem infantil nas áreas de fonologia, vocabulário, fluência e pragmática.

[39] Costa L.M.O., Martins-Reis V.O., Celeste L.C. (2016). Methods of analysis speech rate: a pilot study. CoDAS.

[40] Buzzeti P.B.M.M., Oliveira C.M.C. (2018). Immediate effect of delayed auditory feedback on stuttering-like disfluencies. Rev CEFAC.

[41] Furini J., Picoloto L.A., Marconato E., Bohnen A.J., Cardoso A.C.V., Oliveira C.M.C. (2017). The role of auditory temporal cues in the fluency of stuttering adults. Rev CEFAC.

[42] Arcuri C.F., Osborn E., Schiefer A.M., Chiari B.M. (2009). Speech rate according to stuttering severity. Pró-Fono R Atual Cient.

[43] Chon H., Sawyer J., Ambrose N.G. (2012). Differences of articulation rate and utterance length in fluent and disfluent utterances of preschool children who stutter. J Commun Disord.

[44] Juste F.S., Sassi F.C., Andrade C.R. (2012). Exchange of disfluency with age from function to content words in Brazilian Portuguese speakers who do and do not stutter. Clin Linguist Phon.

[45] Erdemir A., Walden T.A., Jefferson C.M., Choi D., Jones R.M. (2018). The effect of emotion on articulation rate in persistence and recovery of childhood stuttering. J Fluency Disord.

[46] Celeste L.C., Martins-Reis V.O. (2015). The impact of a dysfluency environment on the temporal organization of consoants in stuttering. Audiol Commun Res.

[47] Kikuchi Y., Ogata K., Umesaki T., Yoshiura T., Kenjo M., Hirano Y. (2011). Spatiotemporal signatures of an abnormal auditory system in stuttering. Neuroimage.

[48] Andrade A.N., Gil D., Schiefer A.M., Pereira L.D. (2008). Behavioral auditory processing evaluation in individuals with stuttering. Pró-Fono R Atual Cient.

[49] Silva R., Oliveira C.M.C., Cardoso A.C.V. (2011). Application of temporal pattern tests in children with persistent developmental stuttering. Rev CEFAC.

[50] Arcuri C.F., Schiefer A.M., Azevedo M.F. (2017). Research about suppression effect and auditory processing in individuals who stutter. CoDAS.

